# Mangosteen Peel Liquid-Protected Soybean Meal Can Shift Rumen Microbiome and Rumen Fermentation End-Products in Lactating Crossbred Holstein Friesian Cows

**DOI:** 10.3389/fvets.2021.772043

**Published:** 2022-01-25

**Authors:** Kampanat Phesatcha, Burarat Phesatcha, Metha Wanapat

**Affiliations:** ^1^Department of Animal Science, Faculty of Agriculture and Technology, Nakhon Phanom University, Nakhon Phanom, Thailand; ^2^Department of Agricultural Technology and Environment, Faculty of Sciences and Liberal Arts, Rajamangala University of Technology Isan, Nakhon Ratchasima, Thailand; ^3^Department of Animal Science, Faculty of Agriculture, Tropical Feed Resources Research and Development Center, Khon Kaen University, Khon Kaen, Thailand

**Keywords:** rumen protected protein, microbiome, fermentation, lactating cows, soybean meal (SBM)

## Abstract

Rumen bypass protein can enhance protein availability in the lower gut. This study investigated the use of liquid-containing phytonutrients in dairy cows as a dietary additives to reduce rumen protein degradation. Four crossbred lactating Holstein Friesian cows (75% Holstein Friesian with 25% Thai native breed) with an initial body weight (BW) of 410 ± 20 kg were randomly assigned to a 2 × 2 factorial arrangement [two crude protein (CP) levels with soybean meal (SBM) or mangosteen peel liquid-protected soybean meal (MPLP)-SBM] in a 4 × 4 Latin square design experiment. Dietary treatments were as follows: T1 = SBM in low crude protein concentrate (LPC) (SBM-LPC); T2 = MPLP-SBM in LPC (MPLP-SBM-LPC); T3 = SBM in high crude protein concentrate (HPC) (SBM-HPC); T4 = MPLP-SBM in HPC (MPLP-SBM-HPC). Apparent digestibilities of organic matter (OM) and neutral detergent fiber (aNDF) were increased (*p* < 0.05) by CP level in the HPC diet (19% CP), with higher OM and aNDF digestibilities. High crude protein concentrate increased (*p* < 0.05) the propionic acid in the rumen but reduced (*p* < 0.05) the acetic acid-to-propionic acid ratio and methane (CH_4_) production. Rumen microbial populations of the total bacteria, *Fibrobacter succinogenes* and *Butyrivibrio fibrisolvens* were increased (*p* < 0.05) by HPC. Real-time PCR revealed a 30.6% reduction of rumen methanogens by the MPLP-SBM in HPC. Furthermore, efficiency of microbial nitrogen synthesis (EMNS) was 15.8% increased (*p* < 0.05) by the MPLP-SBM in HPC when compared to SBM-LPC. Milk yield and milk composition protein content were enhanced (*p* < 0.05) by both the CP level in concentrate and by MPLP inclusion. In this experiment, a high level of CP and the MPLP-SBM enhanced the ruminal propionate, shifted rumen microbiome, and enhanced milk yield and compositions.

## Introduction

In the dairy industry, feed cost can contribute about 65–70% of production cost; therefore, improvement of feed utilization efficiency of milk production can have a significant impact on the profitability of dairy cow farming (1). Balancing protein requirement of both rumen-degradable protein (RDP) and rumen-undegradable protein (RUP) could improve the supply of metabolizable protein and reduce the mobilization of the endogenous protein (2, 3). Protein utilization in lactating dairy cattle can improve rumen fermentation and reduce nitrogen loss, which would be beneficial to both animal stockholders and the people (4).

Soybean meal (SBM) is a highly degradable protein in the rumen and has a balance of highly available amino acids (5, 6). Furthermore, SBM is an excellent source of RDP and up to 65% of rumen degradability by rumen microbes (7). However, the excessive degradability of SBM may not be beneficial to high-producing dairy cows, since it increases urine nitrogen loss (6). Some of the treatment methods to reduce rumen degradability of SBM include extrusion, roasting, or expeller or the addition of lignosulfonate, xylose, or formaldehyde and the use of plant secondary compounds. These methods can increase RUP fraction of SBM up to 70% (8, 9). However, the use of phytonutrient to protect protein sources and enhance protein utilization and ruminant performance has very limited information (10, 11). Because of their natural characteristics as compared to chemical additives, phytonutrients condensed tannins (CTs) and saponins (SPs) are important ruminant feed additives, in particular for use as a CH_4_ mitigation strategy and to improve the rumen volatile fatty acid (VFA) profiles (12, 13). Tannins are polyphenolic compounds with high affinity to proteins. Under typical ruminal conditions, these active components can form stable rumen complexes and protect dietary protein from degradation (4, 10). As a consequence of CT supplementation, decreasing protein degradation in the rumen can decrease rumen ammonia nitrogen (NH_3_-N) concentration. However, most of the tannin–protein complexes are required to be dissociated under the acid condition in abomasum, releasing both digestive and absorbent compounds dependent on the binding affinity (14, 15). Naumann et al. (16) stated that CT can decrease CH_4_ synthesis in the rumen either directly or indirectly by decreasing methanogens or protozoal populations, respectively. The CTs have a direct effect on rumen methanogenic archaea by binding the proteinaceous adhesin or parts of the cell envelope, thereby impairing the formation of the methanogen protozoa complex, decreasing interspecies hydrogen transfer, and inhibiting methanogen growth. However, Ku-Vera et al. (17) reported that CTs operate as hydrogen sinks, reducing their availability for carbon dioxide reduction to CH_4_. Makkar and Becker (18) found that SPs have the ability to form complexes with the lipid membranes of bacteria, increasing their permeability, causing an imbalance and, as a result, lysis of the bacterium. The majority of SPs have an effect on protozoa.

Mangosteen (*Garcinia mangostana*) peel is a tropical country agricultural by-product with 16.7% CT and 9.8% SP, which may inhibit some rumen microbes to minimize enteric CH_4_ production. Mangosteen peel as a source of phytonutrients has the potential to be used in feeds for ruminants, with the benefit of reducing the production of CH_4_ and biohydrogenation without adverse effects on ruminal pH and VFA but reduced methanogenic population in the rumen, thus likely reducing the production of CH_4_ in beef cattle and swamp buffaloes (19, 20). The anti-methanogenic activities of CT are linked to a combination of direct toxicity on methanogenic archaea, decreased fiber degradation, or OM digestibility (21, 22). Wanapat et al. (23) stated that mangosteen peel supplementation at 100 g/head/day decreased numbers of methanogens, whereas it increased microbial protein synthesis in swamp buffaloes. Furthermore, Polyorach et al. (24) revealed that mangosteen peel supplementation in lactating dairy cows increased the total bacteria, while the protozoal and methanogen populations decreased. Furthermore, feeding non-protein nitrogen (NPN) such as urea in concentrate may enhance feed intake, digestibility, microbial protein production, and rumen fermentation efficiency, increasing the performance of ruminants fed low-quality roughages (25). Synchronization of ruminal ammonia and energy availability utilizing highly degradable carbohydrates in combination with easily available NPN sources such as urea has been shown to improve the productivity of ruminant though and increased the efficiency of utilization of NH3-N for microbial protein synthesis (25).

Rumen bypass protein is essentially required at the lower gut for digestion, absorption, and utilization by ruminants. A novelty of using mangosteen peel liquid (MPL) containing phytonutrients (CTs and SPs) was developed to protect SBM from rumen degradation and enhance the concentration of protein available in the lower gut. The hypothesis of the experiment was that mangosteen peel liquid-protected soybean meal (MPLP-SBM) could improve the nutrition of feed protein, thus improving milk yield and milk quality in lactating dairy cows. The objective was to determine the effect of MPLP-SBM on rumen fermentation, microbiomes, synthesis of microbial protein, and production of milk yield and composition in lactating dairy cows.

## Materials and Methods

### Preparation of Mangosteen Peel Liquid-Protected Soybean Meal

Fresh mangosteen fruits were purchased from a local market in Muang Khon Kaen, Khon Kaen, Thailand. The fruit pulps were removed, and the peels were dried at 60°C for 48 h and ground to pass through a 1-mm sieve (Cyclotech Mill, Tecator, Hoganas, Sweden). The mangosteen peel meal was mixed with water at 1:5 ratio by adding 1 g of mangosteen peel meal into 5 ml of distillate water. After thorough mixing, it was drained to obtain the mangosteen peel extract solution. The solution was then sprayed onto SBM at 100 g, mixed well by a mixing rotary, and then oven-dried at 60°C for 12 h to obtain the MPLP-SBM. Representative samples of MPLP-SBM were collected and composited, and subsamples and were chemically analyzed for dry matter (DM), organic matter (OM), crude protein (CP), apparent neutral detergent fiber (aNDF), acid detergent fiber (ADF), CT, and SP.

### Animals, Experimental Design, and Dietary Treatments

Four crossbred Holstein Friesian cows (75% Holstein Friesian with 25% Thai native breed) with initial body weight (BW) of 410 ± 20 kg, 125 ± 24 days in milk, and with initial milk yield of 15 ± 5 kg/cow/day were used for the study. The experiment was evaluated in a 4 × 4 Latin square design with four periods, each lasting 21 days, and dietary treatments were as follows: T1 = SBM in low crude protein concentrate (LPC) (SBM-LPC); T2 = MPLP-SBM in LPC (MPLP-SBM-LPC); T3 = SBM in high crude protein concentrate (HPC) (SBM-HPC); T4 = MPLP-SBM in HPC (MPLP-SBM-HPC). The concentrate diet was fed to each cow at the ratio of 1:2 of concentrate to milk yield, while rice straw was *ad libitum*. Feed ingredients and chemical compositions of concentrate and rice straw used in this experiment are presented in Table 1. Cows were kept in individual pens and were fed twice daily at milking time. There was clean fresh water and mineral blocks available all the time. Cows were weighed at the start and at the end of each period.

**Table 1:** Chemical composition of concentrate and rice straw used in the experiment.

**Ingredients**	**Low crude** **protein**		**High crude** **protein**		**Rice straw**
	**–** **MPLP**	**+** **MPLP**	**–** **MPLP**	**+** **MPLP**	
**Feed ingredients**					
Cassava chip	500	500	490	490	
Rice bran	180	180	180	180	
Untreated soybean meal	200	–	200	–	
MPLP soybean meal	–	200	–	200	
Palm kernel meal	70	70	70	70	
Urea	15	15	25	25	
Molasses	5	25	5	25	
Salt	5	5	5	5	
Sulfur	5	5	5	5	
Di-calcium	5	5	5	5	
Premix	5	5	5	5	
**Chemical composition**					
Dry matter, g/kg	916	914	911	913	921
Organic matter	946	958	949	948	893
Crude protein	161	162	191	192	28
Neutral detergent fiber	181	179	175	178	716
Acid detergent fiber	156	154	150	153	514
Condensed tannins	5	18	6	19	-

*MPLP, mangosteen peel liquid-protected treated*.

### Data Collection, Analysis, and Sampling Procedures

Feed, feces, and urine samples were collected during the last 7 days of each period. The feces were collected by rectal sampling, while urine sample was collected by spot sampling following manual stimulation of the vulva to stimulate urination. The urine samples were continuously kept by the addition of sulfuric acid (H_2_SO_4_) to prevent volatilization of NH_3_-N. One hundred milliliters of urine were sampled daily, filtered, and frozen to −18°C for nitrogen analysis later.

The feeds and fecal samples were oven-dried at 60°C; each sample was ground to pass through a 1-mm sieve (Cyclotech Mill, Tecator, Hoganas, Sweden) and was chemically analyzed using the method of AOAC (26) for DM (ID 967.03) and ash (ID 942.05). The ANKOM fiber analyzer (ANKOM A200, ANKOM Technology, NY, USA) was used for determining aNDF and ADF. The ADF content was analyzed according to an AOAC (26) method (ID 967.03) and was expressed inclusive of residual ash; aNDF in samples was estimated according to Van Soest et al. (27) with addition of α-amylase but without sodium sulfite, while acid-insoluble ash was measured according to Van Keulen and Young (28). Total nitrogen (N) was determined according to AOAC (26) (ID 984.13). Content of CT was analyzed by using the modified vanillin-HCl method; SPs were analyzed by using the modified vanillin-sulfuric acid method as described by Wanapat and Poungchompu (29).

Urine samples were chemically analyzed for allantoin and creatinine concentrations using high-performance liquid chromatography (HPLC; instruments by Water and Novapak model 600E; water mode 484 UV detector; column Novapak C_18_; column size 3.9 × 300 mm; Waters Corporation, Milford, MA, USA) and were used to determine microbial purines and total protein of microbes (30). Microbial crude protein (MCP) (g/day) = 3.99 × 0.856 × mmoles of purine derivatives excreted was determined by the method of Galo and Knapp (31). The efficiency of microbial nitrogen synthesis (EMNS) [g of N/kg of organic matter digested in the rumen (OMDR)], assuming that rumen digestion = 65% of OM digestible in total tract (32).

Milk production of all cows was collected every day. Milk samples were randomly collected twice per day in the morning at 5:00 a.m. and in the afternoon at 4:00 p.m. and mixed at 70:30 (from morning and afternoon milk collected) for analysis of fat, protein, lactose, total solids, and solids-not-fat content using Milko-Scan33 (Foss Electric, Hillerod, Denmark). Milk urea nitrogen (MUN) was determined using Sigma kits #640 (Sigma Diagnostics, St. Louis, MO, USA).

On the last day of each sampling period, rumen fluid and jugular blood samples were collected at 0 and 4 h after feeding. Using a stomach tube connected to a vacuum pump, approximately 200 ml of rumen fluid was obtained from the rumen. Rumen fluid was immediately measured for temperature and pH using a portable pH meter (Hanna instrument HI 8424 microcomputer, Pte. Ltd., m 161 Kallang Way, Singapore) and then thoroughly filtered through four layers of cheesecloth. Samples were divided into three portions: first portion of rumen fluid was used for rumen NH_3_-N analysis and total VFA using 5 ml of 1 M H_2_SO_4_ mixed to 45 ml of rumen fluid, which was then centrifuged at 16,000 × *g* for 15 min, and the supernatant was kept at −20°C before NH_3_-N analysis (Kjeltech Auto 1030 Analyzer, Tecator, Höganäs, Sweden). The concentrations of rumen VFA profiles were analyzed using HPLC (instruments by Water and Novapak model 600E; water mode 484 UV detector; column Novapak C_18_; column size 3.9 × 300 mm; mobile phase10 mM H_2_PO_4_, pH 2.5; Waters Corporation, Milford, MA, USA) (33). According to Moss et al. (34), the calculation of ruminal CH_4_ production was based on using VFA proportions as follows: CH_4_ production = 0.45 (C_2_) – 0.275 (C_3_) + 0.4 (C_4_).

A second portion of rumen fluid was kept with 10% formalin solution in sterilized 0.9% saline solution for the total direct counts of protozoa (35). The final portion of rumen fluid was preserved at −20°C for extraction of deoxyribonucleic acid (DNA) (36).

Blood samples were collected from a jugular vein at each sampling time as for rumen fluid and kept into the tubes to which ethylene diamine tetra acetic acid was added. Samples were refrigerated for 1 h and then centrifuged at 3,500 × *g* for 20 min (Table Top Centrifuge PLC-02, Taipei, Taiwan). The plasma was removed and stored at −20°C for the analysis of blood urea nitrogen (BUN) (37).

### Rumen Microbial Population

Community DNA was extracted using Yu and Morrison (36) method. The DNA was purified using columns from QIAgen DNA Mini Stool Kit (QIAGEN, Valencia, CA, USA). Standard PCR conditions for *Fibrobacter succinogenes* were as follows: 30 s at 94°C for denaturing, 30 s at 60°C for annealing, and 30 s at 72°C for extension (48 cycles). For the first cycle, 9-min denaturation time was used. In the last cycle, extension was for 10 min. Amplification of 16S rRNA for *Ruminococcus flavefaciens* and *Ruminococcus albus* was defined in a similar method, with the exception that the annealing temperature was set to 55°C according to Koike and Kobayashi (38). The isolation of genomic DNA was used in real-time quantitative PCR assays with power SYBR Green PCR Master Mix (Applied Biosystems, Warrington, UK), forward and reverse primers, and template DNA. Specified primers were used to measure the microbial population of total bacteria according to Edwards et al. (39), *F. succinogenes, R. flavefaciens*, and *R. albus* (38), *Butyrivibrio fibrisolvens* and *Megasphaera elsdenii* (40), protozoa (41), and methanogenic archaea (42). The DNA standards Real-time PCR amplification and detection were determined using a Chromo 4™ system (Bio-Rad, CA, USA). The data of microbial population were transferred to log10 prior to statistical analysis.

### Statistical Analysis

The results were analyzed in a 4 × 4 Latin square design with 2 × 2 factorial arrangement of treatments with two CP levels (low and high) and two treatments (no treatment and MPLP treatment) using the mixed procedure of the SAS program (43). Data were analyzed using the model:

Yijk = l + Mi + Aj + Pk + eijk

where Yijk is the observation from animal j, receiving diet i, in period k; l, the overall mean; Mi, effect of treatment (i = 1–4); Aj, the effect of animal (j = 1–4); Pk, the effect of period (k = 1–4); and eijk, the residual effect. Significance was declared at *p* < 0.05.

## Results

### Feed Intake and Nutrient Digestibilities

There was no interaction effect between CP levels of concentrate diet and MPLP-SBM on total DM intakes and digestibilities of nutrients as presented in Table 2. The nutrient digestibilities of OM and aNDF were increased (*p* < 0.05) by HPC (19% CP). Digestibility of CP was decreased (*p* < 0.05) when cows were supplemented with MPLP-SBM. In contrast, no effects on digestibilities of DM and ADF by CP level and MPLP-SBM supplementation were found (*p* > 0.05).

**Table 2:** Effect of crude protein level and mangosteen peel liquid-protected soybean meal on voluntary feed intake and apparent digestibility in lactating dairy cows.

**Items**	**Low** **crude protein**		**High** **crude protein**		**SEM**	***p*** **-Value**		
	**–** **MPLP**	**+** **MPLP**	**–** **MPLP**	**+** **MPLP**		**Pro**	**MPLP**	**Pro × MPLP**
**Dry matter intake, kg/day**								
Rice straw	3.4	3.7	3.6	3.7	0.071	0.16	0.56	0.84
Concentrate	8.9	9.5	10.5	11.0	0.584	0.55	0.54	0.58
Total	12.3	13.2	14.1	14.7	0.069	0.13	0.73	0.66
**Apparent digestibility**								
Dry matter	0.641	0.644	0.653	0.662	0.032	0.47	0.09	0.72
Organic matter	0.651	0.652	0.670	0.665	0.014	0.04	0.12	0.65
Crude protein	0.638	0.610	0.642	0.613	0.028	0.10	0.04	0.09
aNeutral detergent fiber	0.581	0.590	0.616	0.609	0.025	0.04	0.09	0.08
Acid detergent fiber	0.442	0.454	0.439	0.456	0.014	0.13	0.51	0.66

*Pro, protein level; MPLP, mangosteen peel liquid-protected treated; Pro × MPLP, protein level × mangosteen peel liquid-protected treated; SEM, standard error of the mean. aNDF, neutral detergent fibraNeutral detergent fiber, amylase-treated neutral detergent fiber*.

### Rumen Fermentation

There was no interaction effect between CP levels of concentrate diet and MPLP-SBM on ruminal pH, NH_3_-N concentration, rumen ecology, total VFA, and VFA profiles (Table 3). Protein levels of concentrate diet and MPLP-SBM supplementation did not reveal any effects on ruminal pH and ruminal temperature in dairy cow. Rumen NH_3_-N concentration and BUN were decreased with MPLP-SBM supplementation (*p* < 0.05). The molar proportions of the respective VFA profiles revealed that there was no interaction between CP level of concentrate diet and MPLP-SBM on C_2_, C_3_, C_4_, and C2:C_3_ ratio. Both CP levels of concentrate diet and MPLP-SBM increased total VFA and C_3_ (*p* < 0.05). However, CP level of concentrate diet decreased (*p* < 0.05) the C_2_:C_3_ ratio, while MPLP-SBM decreased the rumen CH_4_ production (*p* < 0.01).

**Table 3:** Effect of crude protein level and mangosteen peel liquid-protected soybean meal on ruminal fermentation and blood urea nitrogen in lactating dairy cows.

**Items**	**Low** **crude protein**		**High** **crude protein**		**SEM**	***p*** **-Value**		
	**–** **MPLP**	**+** **MPLP**	**–** **MPLP**	**+** **MPLP**		**Pro**	**MPLP**	**Pro × MPLP**
**Ruminal fermentation**								
pH	6.7	6.8	6.8	6.8	0.564	0.62	0.41	0.54
Temperature, °C	39.7	39.6	39.5	39.6	0.311	0.42	0.75	0.18
NH_3_-N, mg/dl	13.9	12.1	17.1	15.0	0.791	0.02	0.04	0.09
Blood urea nitrogen, mg/dl	14.7	12.6	18.1	16.8	0.609	0.04	0.02	0.41
**VFA**								
Total VFA, mmol/l	106.7	110.6	111.6	118.7	0.508	0.04	0.04	0.71
Acetic acid (C_2_), mol/100 mol	64.8	64.4	65.4	64.6	0.971	0.12	0.11	0.88
Propionic acid (C_3_), mol/100 mol	23.9	25.7	24.2	26.0	0.633	0.21	0.02	0.09
Butyric acid (C_4_), mol/100 mol	11.3	9.9	10.4	9.4	0.442	0.32	0.02	0.68
*p*-Value	2.7	2.5	2.7	2.5	0.194	0.14	0.03	0.65
Methane, mM/l^a^	27.1	25.9	26.9	25.6	0.709	0.08	0.03	0.21

*Pro, protein level; MPLP, mangosteen peel liquid-protected treated; Pro × MPLP, protein level × mangosteen peel liquid-protected treated; SEM, standard error of the mean.*

a *Calculated according to Moss et al. (34).*

*Methane production = 0.45 (acetate) – 0.275 (propionate) + 0.4 (butyrate)*.

### Ruminal Microbes

Table 4 shows the effect of CP level and/or MPLP-SBM supplementation on the rumen microorganism population. There was no interaction effect between CP level in concentrate diet and MPLP-SBM supplementation on rumen microorganism population. Moreover, total bacteria, *F. succinogenes* and *B. fibrisolvens* populations were increased by both higher CP level in concentrate and presence of MPLP-SBM (*p* < 0.05). Methanogens and protozoal population were decreased when cows were supplemented with MPLP-SBM (*p* < 0.05).

**Table 4:** Effect of crude protein level and mangosteen peel liquid-protected soybean meal on rumen microbial population in lactating dairy cows.

**Items**	**Low** **crude protein**		**High** **crude protein**		**SEM**	***p*** **-Value**		
	**–** **MPLP**	**+** **MPLP**	**–** **MPLP**	**+** **MPLP**		**Pro**	**MPLP**	**Pro × MPLP**
**Direct count, cell/ml**								
Protozoa, × 10^5^	7.2	4.9	7.8	5.3	0.981	0.642	0.019	0.438
**Real-time PCR, copies/ml rumen content**								
Total bacteria, × 10^8^	0.7	4.6	7.7	9.8	0.210	0.02	0.02	0.33
*F. succinogenes*, × 10^7^	2.6	3.4	6.8	7.5	0.135	0.02	0.02	0.41
*R. flavefaciens*, × 10^6^	4.7	4.4	5.7	5.2	0.047	0.72	0.51	0.22
*R. albus*, × 10^7^	3.5	4.1	4.6	5.4	0.065	0.68	0.31	0.42
*B. fibrisolvens*, × 10^6^	2.1	3.2	5.5	6.1	0.081	0.02	0.02	0.12
*M. eleinii*, × 10^7^	3.5	4.1	4.6	5.4	0.042	0.36	0.05	0.71
Methanogens, × 10^3^	5.7	3.2	4.9	3.4	0.059	0.41	0.03	0.16
Protozoa, × 10^4^	4.8	3.2	5.1	3.6	0.198	0.71	0.03	0.53

*Pro, protein level; MPLP, mangosteen peel liquid-protected treated; Pro × MPLP, protein level × mangosteen peel liquid-protected treated; SEM, standard error of the mean*.

### Microbial Protein Synthesis

There was no interaction effect between CP level in concentrate diet and MPLP-SBM supplementation on purine derivative, MCP, and EMNS, as shown in Table 5. The excretion of allantoin was not altered in any treatment (*p* > 0.05). However, the absorption of allantoin and MCP were increased by MPLP-SBM supplementation (*p* < 0.05). The allantoin absorption ranged from 261.4 to 315.2 g/day. In addition, the EMNS was increased in cows supplemented with MPLP-SBM (*p* < 0.05).

**Table 5:** Effect of crude protein level and mangosteen peel liquid-protected soybean meal on microbial protein synthesis in lactating dairy cows.

**Items**	**Low** **crude protein**		**High** **crude protein**		**SEM**	***p*** **-Value**		
	**–** **MPLP**	**+** **MPLP**	**–** **MPLP**	**+** **MPLP**		**Pro**	**MPLP**	**Pro × MPLP**
Purine derivatives, mmol/day								
Allantoin excretion	236.9	254.4	261.9	272.7	3.198	0.43	0.71	0.42
Allantoin absorption	261.4	283.1	287.7	315.2	2.654	0.07	0.04	0.06
Urine creatinine	26.2	26.5	27.3	28.6	0.614	0.06	0.05	0.05
MCP^a^, g/day	702.4	720.3	735.7	764.6	1.637	0.03	0.01	0.21
EMNS^b^, g/kg OMDR	25.9	27.1	28.4	32.9	0.879	0.04	0.01	0.41

*Pro, protein level; MPLP, mangosteen peel liquid-protected treated; Pro × MPLP, protein level × mangosteen peel liquid-protected treated, SEM, standard error of the mean.*

a *Microbial crude protein (MCP) (g/day) = 3.99 ×0.856 × mmol of purine derivatives excreted (31).*

b *Efficiency of microbial nitrogen supply (EMNS), g/kg of organic matter (OM) digested in the rumen (OMDR) = {[MCP (g/day) × 1,000]/DOMR (g)}, assuming that rumen digestion = 65% of digestion in total tract*.

### Milk Yield and Compositions

There was no significant interaction effect between CP level in concentrate diet and MPLP-SBM supplementation on milk yield and compositions (Table 6). Milk yield and 3.5% FCM yield were found the highest in cows supplemented with MPLP-SBM in concentrate containing high crude protein. Moreover, milk compositions including fat, lactose, solids-not-fat, total solids, and MUN were not affected (*p* > 0.05) by CP level and MPLP-SBM supplementation, but milk protein was increased (*p* < 0.05) in the high crude protein level in concentrate diet.

**Table 6:** Effect of crude protein level and mangosteen peel liquid-protected soybean meal on milk yield and composition in lactating dairy cows.

**Items**	**Low** **crude protein**		**High** **crude protein**		**SEM**	***p*** **-Value**		
	**–** **MPLP**	**+** **MPLP**	**–** **MPLP**	**+** **MPLP**		**Pro**	**MPLP**	**Pro × MPLP**
Production								
Milk yield, kg/day	17.8	19.2	21.8	23.5	0.886	0.039	0.04	0.41
3.5% FCM, kg/day	18.6	21.1	24.3	26.2	0.879	0.010	0.04	0.49
Fat	3.8	4.1	4.2	4.2	0.418	0.195	0.29	0.61
Protein	3.1	3.4	3.7	3.8	0.154	0.020	0.13	0.07
Lactose	4.6	4.7	4.7	4.7	0.104	0.187	0.36	0.12
Solids non-fat	9.2	9.1	9.2	9.3	0.207	0.081	0.45	0.33
Total solids	13.8	14.1	14.3	14.0	0.388	0.652	0.51	0.21
Milk urea N, mg/dl	12.4	12.6	12.8	13.1	0.547	0.174	0.19	0.29

*Pro, protein level; MPLP, mangosteen peel liquid-protected treated; Pro × MPLP, protein level × mangosteen peel liquid-protected treated; SEM, standard error of the mean; FCM, fat-corrected milk*.

## Discussion

### Feed Intake and Nutrient Digestibilities

Dietary CP concentration in ruminant rations is an important factor supporting growth and lactation. Colmenero and Broderick (44) reported that increased level of CP resulted in an improvement of CP and ADF digestibilities (*p* < 0.05), while Dung et al. (45) found that HPC increased CP digestibility but had no effect on DM, OM, and NDF digestibilities. In the present study, the nutrient digestibilities of OM and aNDF were increased (*p* < 0.05) by the HPC (19% CP).

Mangosteen peel liquid used in this study contained CT 16.9% and SP 9.6%, which was comparable to that of Wanapat et al. (23) who reported that mangosteen peel contained CT 17.9% and SP 9.2%. Under this trial, MPLP-SBM did not change the DM intakes. Manasri et al. (46) found when supplemented CT from mangosteen peel at 0.12 g/head/day did not have an effect on DM intake in beef cattle, while Polyorach et al. (24) also found supplementation of MSP at 300 g/head/day with YEFECAP as a protein source in concentrate mixture remarkably improved the microbial fermentation efficiency. Supplementation of mangosteen peel at 300 g/head/day remarkably improved the microbial fermentation efficiency in dairy cows. However, when high CT was used, the apparent digestibility of nutrients was reduced (*p* < 0.05) (4, 19). Dschaak et al. (47) showed that the supplementation of 3% quebracho CT resulted in reduced DM intake, while the production of milk and milk composition were unchanged. The variety and concentration of phytonutrients of the plant may also be due to many effects including type and growth stages. There were no significant interaction effects of the two factors found on DM intakes and nutrient digestibilities under this experiment.

### Rumen Fermentation

Ruminal temperature and pH remained unchanged among the dietary treatments, and both were in optimum range (pH 6.5–7.0) especially for fiber digestion (48). In the present study, increasing the dietary CP level increased (*p* < 0.05) the concentration of NH_3_-N. Similarly, Xia et al. (49) reported that the NH_3_-N concentration of the bulls receiving the high crude protein level was significantly higher than those receiving the low crude protein diet. In contrast, Bahrami-Yekdangi et al. (50) observed that the concentration of NH_3_-N diet was unchanged.

The concentration of NH_3_-N was reduced (*p* < 0.05) by MPLP-SBM. This could be due to increased microbial protein synthesis that can consequently reduce the NH_3_-N concentration in the rumen. However, CTs have a high capacity for CP binding in the rumen and would reduce dietary protein loss by ammonia production, thus improving protein utilization (22). El-Waziry et al. (51) found that the concentration of ruminal NH_3_-N was decreased with protected SBM. Reducing NH_3_-N in the rumen means that treated SBM reduced peptide degradation, proteolysis, and amino acid deamination in the rumen (52). Both factors exhibited significant effects, but there were no interactions. Ampapon et al. (20) stated that plant phytonutrients such as CT exhibited selective suppression of cellulolytic bacteria in the rumen. Due to the complexation of protein–tannin, MPLP-SBM supplementation in the diet has beneficial effects, reducing the availability of feed protein for possible ruminal degradation to produce ammonia–nitrogen. The concentrations of total VFA and C_3_ were increased (*p* < 0.05) by protein level or by MPLP inclusion. Wanapat et al. (23) reported that the mangosteen peel supplementation in buffaloes impacted total rumen VFA production and increased the C_3_ concentration, while reducing C_2_:C_3_ and the production of CH_4_. Additionally, Polyorach et al. (24) also demonstrated that the use of CT from mangosteen powder could increase total VFA concentration especially C_3_. Moreover, under the present study, CH_4_ production was reduced (*p* < 0.05) by MPLP-SBM supplementation. These results could be due to the suppression of methanogens that adhered to protozoa in the rumen for additional activity. Poungchompu et al. (19) revealed that plants containing CT and SP greatly improved ruminal feed degradation by mitigating the C_2_ concentration and CH_4_ production, hence enhancing the C_3_ concentration. Recently, Ampapon et al. (20) revealed that supplementing mangosteen peel could reduce the production of CH_4_ by suppressing ruminal protozoa. However, the results of mangosteen peel supplementation did not change total VFA and individual VFA as reported by Ngamsaeng et al. (53). Many previous studies have stated that CT and SP or their extracts were effective in reducing CH_4_ production in both *in vitro* and *in vivo* studies (54, 55). Only the MPLP supplementation resulted in a reduction (*p* < 0.05) of C2:C3 ratio and CH_4_ production, and no interactions were found.

### Ruminal Microbes

The populations of cellulolytic bacteria, *F. succinogenes* and *B. fibrisolvens* were increased (*p* < 0.05), while those of *M. elsdenii* were not changed (*p* > 0.05) by the MPLP-SBM. The populations of *R. albus* and *R. flavefaciens* were not changed while methanogen was decreased (*p* < 0.05) when cows were supplemented with MPLP-SBM. Dong et al. (56) found that *Moringa oleifera* containing CT altered the composition and diversity of methanogens, hence mitigating CH_4_ emissions in dairy cows. However, Anantasook et al. (57) revealed that the population of *F. succinogenes* was increased when dairy cows were fed with *Samanea saman* (rain tree pod meal) containing CT and SP possibly as an effect of suppression of protozoa and methanogen numbers. Norrapoke et al. (58) described the fibrolytic bacteria species in the rumen and noted that *R. flavefaciens, R. albus*, and *F. succinogenes* to a higher extent degraded crystalline cellulose more effectively than the ruminococcal species (38). In the present study, methanogens were significantly reduced (*p* < 0.05) when dairy cows were supplemented with MPLP-SBM. Wanapat et al. (23) stated that mangosteen peel supplementation at 100 g/head/day in swamp buffaloes increased the total number of rumen bacteria and *R. flavefaciens*, while methanogens were decreased (*p* < 0.05). Both factors under this investigation showed significant effects on total bacteria, *F. succinogenes* and *B. fibrisolvens*, but no interaction effects between the CP level and MPLP-SBM were obtained.

### Microbial Protein Synthesis

In the present study, CP in concentrate diet and MPLP-SBM did not affect allantoin excretion and urine creatinine in milking dairy cows. Zhang et al. (4) stated that CT supplementation in milking dairy cows altered the N excretion route, led to less urinary N excretion, but failed to alter the N utilization efficiency for milk production. Under this work, only a high level of CP in concentrate impacted MCP. Most of the proteins supplied to the small intestine of ruminants could be supplied by microbial protein synthesis in the rumen, comprising 50–80% of overall absorbable protein (59), while MCP in the present study ranged from 702.4 to 764.6 g/day. Furthermore, high crude protein supplementation with MPLP-SBM inclusion resulted in the highest MCP among the treatments. Wanapat et al. (23) noted that when total dietary N intake was low, urea supplementation enhanced the EMNS. Higher amount of urea recycling could enhance microbial protein synthesis, hence the EMNS, accordingly (60). Wanapat et al. (23) stated that ruminal microbial CP synthesis is primarily dependent on the sufficiency of carbohydrates as an energy source and the available NPN. Also, lactating dairy cows supplemented with CT resulted in improved EMNS (24). Both the ruminal dietary protein degradation and the synchrony of nitrogen and energy balance will affect the effectiveness of any dietary supplement, including CT supplements, on the EMNS (61, 62). Among the parameters MCP and EMNS were significantly increased by both factors, and no interactions were found.

### Milk Yield and Compositions

In the present study, increasing the dietary CP level resulted in improved milk yield. This finding is consistent with Law et al. (63) who found increases in milk yield as dietary CP was increased. However, Xia et al. (49) revealed that milk yield did not differ across CP levels in the diet.

Milk yield and milk protein were significantly increased with the supplementation of MPLP-SBM. The CT's MPLP-SBM content may interact with SBM to produce a CT–protein bound complex that would prevent the digestion of dietary protein in the rumen with higher dietary protein flow to the duodenum that could be achieved for the dairy cows (21). Anantasook et al. (57) stated that supplementation with CT from rain tree pod meals resulted in a higher milk protein and solids non-fat when compared with the control. Broderick et al. (64) suggested that silage containing 0.5% of CT improved milk yield and milk protein. Tannins have the potential to increase RUP in a diet, and when combined with high-quality protein that contains a significant amount of limiting amino acid like methionine and lysine, this could lead to an improvement in N efficiency and milk production by increasing the flow of an adequate supply of amino acid to the small intestine (11). In addition, Polyorach et al. (24) found that mangosteen peel supplementation could enhance DM intake and digestibility of nutrients and improve the fermentation process, milk yield, and composition in lactating dairy cows. However, Benchaar et al. (65) noted that when plants rich in tannins were supplemented in the diets of dairy cows, milk composition remains unchanged. Both factors resulted in significant enhancement of milk yield and 3.5% FCM, but there were no interactions found.

Blood urea nitrogen has been shown to be an indicator of the metabolism of nitrogen in ruminants, and higher BUN can imply greater degradation of the ruminal protein (66). The concentration of BUN and MUN were not changed among treatments, and the values were in the normal range. Roseler et al. (67) and Jonker et al. (68) reported the normal ranges of 15 mg/dl for MUN and 5–6 mg/dl for BUN.

## Conclusions

High crude protein level in concentrate and MPLP-SBM supplementation enhanced rumen C_3_, but reduced C_2_:C_3_ ratio and CH_4_ production. Total bacterial numbers and *F. succinogenes* population were increased (*p* < 0.05) with high protein level in concentrate and MPLP-SBM supplementation. Allantoin absorption was higher with MPLP-SBM supplementation, while protein content influenced MCP and EMNS with higher values (*p* < 0.05) in the high CP level in concentrate. Importantly, milk yield and 3.5% FCM were remarkably enhanced (*p* < 0.05) by the MPLP-SBM with HPC. Hence, it is recommended that MPLP treatment of SBM should be exploited as a feeding strategy to improve milk yield and compositions in lactating dairy cows.

## Data Availability Statement

The original contributions presented in the study are included in the article/supplementary material, further inquiries can be directed to the corresponding author/s.

## Ethics Statement

The animal study was reviewed and approved by the Khon Kaen University Animal Ethics Committee.

## Author Contributions

KP and MW designed the research. KP and BP conducted the research. KP analyzed the data and wrote the manuscript. All authors approved the final manuscript.

## Conflict of Interest

The authors declare that the research was conducted in the absence of any commercial or financial relationships that could be construed as a potential conflict of interest.

## Publisher's Note

All claims expressed in this article are solely those of the authors and do not necessarily represent those of their affiliated organizations, or those of the publisher, the editors and the reviewers. Any product that may be evaluated in this article, or claim that may be made by its manufacturer, is not guaranteed or endorsed by the publisher.

## Abbreviations

ADF: acid detergent fiber
BUN: blood urea nitrogen
BW: body weight
C_2_: acetic acid
C_3_: propionic acid
C_4_: butyric acid
CH_4_: methane
CP: crude protein
CT: condensed tannins
DM: dry matter
EMNS: efficiency of microbial nitrogen supply
FCM: fat-corrected milk
HPC: high crude protein concentrate
LPC: low crude protein concentrate
MCP: microbial crude protein
MPLP-SBM: mangosteen peel liquid-protected soybean meal
aNDF: apparent neutral detergent fiber
NH_3_-N: ammonia nitrogen
OM: organic matter
OMDR, organic matter digested in the rumen, MUN: milk urea nitrogen
PCR: polymerase chain reaction
RDP: rumen-degradable protein
RUP: rumen-undegradable protein
SBM: soybean meal
SP: saponins
TVFA: total volatile fatty acid.

## References

[1] Jolazadeh A Dehghan-banadaki RM Rezayazdi K. Effects of soybean meal treated with tannins extracted from pistachio hulls on performance, ruminal fermentation, blood metabolites and nutrient digestion of Holstein bulls. Anim Feed Sci Technol. (2015) 203:33–40. 10.1016/j.anifeedsci.2015.02.005

[2] Costa JBG Zeoula LM Franco SL Moura LPP Valero MV Simioni FL. Effect of propolis product on digestibility and ruminal parameters in buffaloes consuming a forage-based diet. Ital J Anim Sci. (2012) 11:4. 10.4081/ijas.2012.e78

[3] Rufino LM Detmann E Gomez DI Reis WL Batista ED Valadares SC. Intake, digestibility and nitrogen utilization in cattle fed tropical forage and supplemented with protein in the rumen, abomasum, or both. J Anim Sci Biotechnol. (2016) 7:11. 10.1186/s40104-016-0069-9 26900467 PMC4761222

[4] Zhang J Zu X Cao Z Li S Wang Y Yang H. Effects of different tannin sources on nutrient intake, digestibility, performance, nitrogen utilization, and blood parameters in dairy cows. Animals. (2019) 9:507. 10.3390/ani9080507 31370306 PMC6719915

[5] Gidlund H Hetta M Krizsan SJ Lemosquet S Huhtanen P. Effects of soybean meal or canola meal on milk production and methane emissions in lactating dairy cows fed grass silage-based diets. J Dairy Sci. (2015) 98:8093–106. 10.3168/jds.2015-9757 26364100

[6] Wang Z Yu Y Li X Xiao H Zhang P Shen W. Fermented soybean meal replacement in the diet of lactating Holstein dairy cows: modulated rumen fermentation and ruminal microflora. Front Microbiol. (2021) 12:625857. 10.3389/fmicb.2021.625857 33584627 PMC7879537

[7] NRC. Nutrient Requirements of Dairy Cattle. 7th ed. Washington, DC: National Academy Press (2001).

[8] McMahon LR McAllister TA Berg BP Majak W Acharya SN Popp JD. A review of the effects of forage condensed tannins on ruminal fermentation and bloat in grazing cattle. Can J Plant Sci. (2000) 80:469–85. 10.4141/P99-050

[9] Kazemi-Bonchenari M Alizadeh AR Tahriri AR Karkoodi K Jalali S Sadri H. The effects of partial replacement of soybean meal by xylose-treated soybean meal in the starter concentrate on performance, health status, and blood metabolites of Holstein calves. Ital J Anim Sci. (2015) 14:138–42. 10.4081/ijas.2015.3680

[10] Focant M Froidmont E Dang Van QC Larondell Y. The effect of oak tannin (*Quercus robur*) and hops (*Humulus lupulus*) on dietary nitrogen efficiency, methane emission, and milk fatty acid composition of dairy cows fed a low-protein diet including linseed. J Dairy Sci.(2019) 102:1144–59. 10.3168/jds.2018-15479 30594358

[11] Grazziotin RCB Halfen J Rosa F Schmitt E Anderson JL Ballard V. Altered rumen fermentation patterns in lactating dairy cows supplemented with phytochemicals improve milk production and efficiency. J Dairy Sci. (2020) 103:301–12. 10.3168/jds.2019-16996 31733851

[12] Wanapat M Kang S Polyorach S. Development of feeding systems and strategies of supplementation to enhance rumen fermentation and ruminant production in the tropics. J Anim Sci Biotechnol. (2013) 4:32. 10.1186/2049-1891-4-32 23981662 PMC3765718

[13] Dang Van QC Gardin C Mignolet E Froidmont E Focant M Larondelle Y. *In vitro* effects of hop pellets and oak extracts in combination on ruminal fermentation parameters. Biotechnol Agron Soc Environ. (2018) 22:1–9. 10.25518/1780-4507.16370

[14] Patra AK Saxena J. Exploitation of dietary tannins to improve rumen metabolism and ruminant nutrition. J Sci Food Agric. (2011) 91:24–37. 10.1002/jsfa.4152 20815041

[15] Henke A Dickhoefer U Westreicher-Kristen E Knappstein K Molkentin J Hasler M. Effect of dietary quebracho tannin extract on milk fatty acid composition in cows. Arch Anim Nutr.(2017) 17:37–53. 10.1080/1745039X.2016.1250541 27830586

[16] Naumann HD Tedeschi LO Zeller WE Huntley NF. The role of condensed tannins in ruminant animal production: advances, limitations and future directions. Rev Brasil Zootec. (2017) 46:929–49. 10.1590/s1806-92902017001200009

[17] Ku-Vera JC Jiménez-Ocampo R Valencia-Salazar SS Montoya-Flores MD Molina-Botero IC Arango J. Role of secondary plant metabolites on enteric methane mitigation in ruminants. Front Vet Sci. (2020) 7:584. 10.3389/fvets.2020.00584 33195495 PMC7481446

[18] Makkar HPS Blümmel M Becker K. *In vitro* effects of and interactions between tannins and saponins and fate of tannins in the rumen. J Sci Food Agric. (1995) 69:481–93. 10.1002/jsfa.274069041325855820

[19] Poungchompu O Wanapat M Wachirapakorn C Wanapat S Cherdthong A. Manipulation of ruminal fermentation and methane production by dietary saponins and tannins from mangosteen peel and soapberry fruit. Arch Anim Nutr. (2009) 63:389–400. 10.1080/17450390903020406 26967797

[20] Ampapon T Phesatcha K Wanapat M. Effects of phytonutrients on ruminal fermentation, digestibility, and microorganisms in swamp buffaloes. Animals. (2019) 9:671. 10.3390/ani9090671 31514374 PMC6770294

[21] Patra AK Saxena J. A new perspective on the use of plant secondary metabolites to inhibit methanogenesis in the rumen. Phytochem. (2010) 71:1198–222. 10.1016/j.phytochem.2010.05.010 20570294

[22] Adejoro FA Hassen A Thantsha MS. Preparation of acacia tannin loaded lipid microparticles by solid-in-oil-in-water and melt dispersion methods, their characterization and evaluation of their effect on ruminal gas production *in vitro*. PLoS ONE. (2018) 13:e0206241. 10.1371/journal.pone.0206241 30359451 PMC6201938

[23] Wanapat M Chanthakhoun V Phesatcha K Kang S. Influence of mangosteen peel powder as a source of plant secondary compounds on rumen microorganisms, volatile fatty acids, methane and microbial protein synthesis in swamp buffaloes. Livest Sci. (2014) 162:126–33. 10.1016/j.livsci.2014.01.025

[24] Polyorach S Wanapat M Cherdthong A Kang S. Rumen microorganisms, methane production, and microbial protein synthesis affected by mangosteen peel powder supplement in lactating dairy cows. Trop Anim Health Prod. (2016) 48: 593–601. 10.1007/s11250-016-1004-y 26885988

[25] Kang S Wanapat M Phesatcha K Norrapoke T. Effect of protein level and urea in concentrate mixture on feed intake and rumen fermentation in swamp buffaloes fed rice straw-based diet. Trop Anim Health Prod. (2015) 47:671–9. 10.1007/s11250-015-0777-8 25686554

[26] AOAC. Official Methods of Analysis, 16th ed. Off. Anal. Chem., Arlington, VA: AOAC (1995).

[27] Van Soest PJ Robertson JB Lewis BA. Methods for dietary fiber, neutral detergent fiber, and non-starch polysaccharides in relation to animal nutrition. J Dairy Sci. (1991) 74:3583–97. 10.3168/jds.S0022-0302(91)78551-2 1660498

[28] Van Keulen JY Young BA. Evaluation of acid-insoluble ash as a natural marker in ruminant digestibility studies. J Anim Sci. (1977) 44:282–7. 10.2527/jas1977.442282x

[29] Wanapat M Poungchompu O. Method for Estimation of Tannin by Vanillin-HCL Method (A Modified Method of Burns, 1971). Department of Animal Science, Khon Kaen University, Khon Kaen, Thailand (2001).

[30] Chen XB Gomes MJ. Estimation of Microbial Protein Supply to Sheep and Cattle Based on Urinary Excretion of Purine Derivatives - An Overview of Technical Details. Rowett Research Institute, Aberdeen, UK: International Feed Resources Unit, Occasional Publication (1992).

[31] Galo E Emanuele SM Sniffen CJ White JH Knapp JR. Effects of a polymer-coated urea product on nitrogen metabolism in lactating Holstein dairy cattle. J Dairy Sci. (2003) 86:2154–62. 10.3168/jds.S0022-0302(03)73805-3 12836952

[32] Agricultural Research Council. The Nutrient Requirements of Ruminant Livestock. Farnham Royal. UK Commonwealth Agricultural Bureaux (1980).

[33] Samuel M Sagatheman S Thomas J Mathen G. An HPLC method for estimation of volatile fatty acids of ruminal fluid. Indian J Anim Sci. (1997) 67:805–7.

[34] Moss AR Jouany JP Newbold J. Methane production by ruminants: its contribution to global warming. Annal Zootech. (2000) 49:231–53. 10.1051/animres:200011920400576

[35] Galyean M. Laboratory Procedure in Animal Nutrition Research. Department of Animal and Range Sciences, New Mexico State University, Las Cruces, NM (1989).

[36] Yu Z Morrison M. Improved extraction of PCR quality community DNA from digesta and fecal samples. Bio Techniques. (2004) 36:808–12. 10.2144/04365ST04 15152600

[37] Crocker CL. Rapid determination of urea nitrogen in serum or plasma without deproteinization. Am J Med Technol. (1967) 33:361. 6056194

[38] Koike S Kobayashi Y. Develop and use of competitive PCR assays for the rumen cellulolytic bacteria: *Fibrobactor succinogenes, Ruminococcus albus* and *Ruminococcus flavefaciens*. FEMS Microbiol Ecol. (2001) 204:361–6. 10.1111/j.1574-6968.2001.tb10911.x 11731149

[39] Edwards JE Huws SA Kim EJ Kingston-Smith AH. Characterization of the dynamics of initial bacterial colonization of nonconserved forage in the bovine rumen. FEMS Microbiol Ecol. (2007) 62:323–35. 10.1111/j.1574-6941.2007.00392.x 17941835

[40] Stevenson DM Weimer PJ. Dominance of prevotella and low abundance of classical ruminal bacterial species in the bovine rumen revealed by relative quantification real-time PCR. Appl Microbiol Biotechnol. (2007) 75:165–74. 10.1007/s00253-006-0802-y 17235560

[41] Sylvester JT Karnati SKR Yu Z Morrison M Firkins JL. Development of an assay to quantify rumen ciliate protozoal biomass in cows using real-time PCR. J Nutr. (2004) 134:3378–84. 10.1093/jn/134.12.3378 15570040

[42] Denman SE Tomkins NW McSweeney CS. Quantitation and diversity analysis of ruminal methanogenic populations in response to the antimethanogenic compound bromochloromethane. FEMS Microbiol Ecol. (2007) 62:313–22. 10.1111/j.1574-6941.2007.00394.x 17949432

[43] SAS Institute Inc. SAS/STAT(r)9.2 User's Guide. SAS Inst. Inc., Cary, NC (2009).

[44] Colmenero JJO Broderick GA. Effect of dietary crude protein concentration on milk production and nitrogen utilization in lactating dairy cows. J Dairy Sci. (2006) 89:1704–12. 10.3168/jds.S0022-0302(06)72238-X 16606741

[45] Dung DV Ba NX Van NH. Practice on improving fattening local cattle production in Vietnam by increasing crude protein level in concentrate and concentrate level. Trop Anim Health Prod. (2013) 45:1619–26. 10.1007/s11250-013-0407-2 23619780

[46] Manasri N Wanapat M Navanukraw C. Improving rumen fermentation and feed digestibility in cattle by mangosteen peel and garlic pellet supplementation. Livest Sci. (2012) 148:291–5. 10.1016/j.livsci.2012.06.009

[47] Dschaak CM Williams CM Holt MS Eun JS Young AJ Min BR. Effects of supplementing condensed tannin extract on intake, digestion, ruminal fermentation, and milk production of lactating dairy cows. J Dairy Sci. (2011) 94:2508–19. 10.3168/jds.2010-3818 21524543

[48] Zicarelli F Calabrò S Cutrignelli MI Infascelli F Tudisco R Bovera F. *In vitro* fermentation characteristics of diets with different forage/concentrate ratios: comparison of rumen and faecal inocula. J Sci Food Agric. (2011) 91:1213–1221. 10.1002/jsfa.4302 21360539

[49] Xia C Rahman MZU Yang H Shao T Qiu Q. Effect of increased dietary crude protein levels on production performance, nitrogen utilization, blood metabolites and ruminal fermentation of Holstein bulls. Asian-Australas J Anim Sci. (2018) 31:1643–53. 10.5713/ajas.18.0125 29879836 PMC6127588

[50] Bahrami-Yekdangi M Ghorbani GR Khorvash M Khan MA Ghaffari MH. Reducing crude protein and rumen degradable protein with a constant concentration of rumen undegradable protein in the diet of dairy cows: production performance, nutrient digestibility, nitrogen efficiency, and blood metabolites. J Anim Sci. (2016) 94:718–25. 10.2527/jas.2015-9947 27065142

[51] El-Waziry AM AlKoaik F Khalil AI Metwally H AlMahasneh MA. Estimation of degradability kinetics, energy and organic matter digestibility of date palm (*Phoenix dactylifera* L.) leaves silage by *in vitro* gas production technique. Asian J Anim Vet Adv. (2013) 8:814–20. 10.3923/ajava.2013.814.820

[52] Newbold CJ Hassan SM Wang J Ortega ME Wallace RJ. Influence of foliage from African multipurpose trees on activity of rumen protozoa and bacteria. Br J Nutr. (1997) 78:237–49. 10.1079/BJN19970143 9301414

[53] Ngamsaeng A Wanapat M Khampa S. Effects of mangosteen peel (*Garcinia mangostana* L.) supplementation on rumen ecology, microbial protein synthesis, digestibility and voluntary feed intake in cattle. Pak J Nutr.(2006) 5:445–52. 10.3923/pjn.2006.445.452

[54] Abdalla AL Louvandini H Sallam SM Bueno IC Tsai SM Figueira AV. *In vitro* evaluation, *in vivo* quantification, and microbial diversity studies of nutritional strategies for reducing enteric methane production. Trop Anim Health Prod. (2012) 44:953–64. 10.1007/s11250-011-9992-0 22083272

[55] Meale SJ Chaves AV Baah J McAllister TA. Methane production of different forages in *in vitro* ruminal fermentation. Asian Australas J Anim Sci. (2012) 25:86–91. 10.5713/ajas.2011.11249 25049482 PMC4092917

[56] Dong L Zhang T Diao Q. Effect of dietary supplementation of *Moringa oleifera* on the production performance and fecal methanogenic community of lactating dairy cows. Animals (Basel). (2019) 9:262. 10.3390/ani9050262 31121857 PMC6562924

[57] Anantasook N Wanapat M Cherdthong A Gunun P. Effect of tannins and saponins in *Samanea saman* on rumen environment, milk yield and milk composition in lactating dairy cows. J Anim Physiol Anim Nutr. (2015) 99:335–334. 10.1111/jpn.12198 24814291

[58] Norrapoke T Wanapat M Wanapat S. Effects of protein level and mangosteen peel pellets (mago-pel) in concentrate diets on rumen fermentation and milk production in lactating dairy crossbreds. Asian Australas J Anim Sci. (2012) 25:971–9. 10.5713/ajas.2012.12053 25049652 PMC4092972

[59] Firkins JL Yu Z Morrison M. Ruminal nitrogen metabolism: perspectives for integration of microbiology and nutrition for dairy. J Dairy Sci. (2007) 90:1–16. 10.3168/jds.2006-518 17517749

[60] Jouany JP Morgavi DP. Use of ‘natural' products as alternatives to antibiotic feed additives in ruminant production. Animals. (2007) 1:1443–66. 10.1017/S1751731107000742 22444918

[61] McSweeney CS Palmer B McNeill DM Krause DO. Microbial interactions with tannins: Nutritional consequences for ruminants. Anim Feed Sci Technol. (2001) 91:83–93. 10.1016/S0377-8401(01)00232-2

[62] Greenwood SL Edwards GR Harrison R. Short communication: Supplementing grape marc to cows fed a pasture-based diet as a method to alter nitrogen partitioning and excretion. J Dairy Sci. (2012) 95:755–8. 10.3168/jds.2011-4648 22281340

[63] Law RA Young FJ Patterson DC Kilpatrick DJ Wylie ARG Mayne CS. Effect of dietary protein content on animal production and blood metabolites of dairy cows during lactation. J Dairy Sci. (2009) 92:1001–12. 10.3168/jds.2008-1155 19233794

[64] Broderick GA Grabber JH Muck RE Hymes-Fecht UC. Replacing alfalfa silage with tannin-containing birds foot trefoil silage in total mixed rations for lactating dairy cows. J Dairy Sci. (2017) 100:3548–3562. 10.3168/jds.2016-12073 28259401

[65] Benchaar C McAllister TA Chouinard PY. Digestion, ruminal fermentation, ciliate protozoal populations, and milk production from dairy cows fed cinnamaldehyde, quebracho condensed tannin, or *Yucca schidigera* saponin extracts. J Dairy Sci. (2008) 91:4765–77. 10.3168/jds.2008-1338 19038952

[66] Broderick GA Clayton MK. A statistical evaluation of animal and nutritional factors influencing concentrations of milk urea nitrogen. J Dairy Sci. (1997) 80:2964–71. 10.3168/jds.S0022-0302(97)76262-3 9406089

[67] Roseler DK Ferguson JD Sniffen CJ Herrema J. Dietary protein degradability effects on plasma and milk urea nitrogen and milk non protein in Holstein cow. J Dairy Sci. (1993) 76:525–34. 10.3168/jds.S0022-0302(93)77372-5

[68] Jonker JS Kohn RA Erdman RA. Milk urea nitrogen target concentrations for lactating dairy cows fed according to national research council recommendations. J Dairy Sci. (1999) 82:1261–73. 10.3168/jds.S0022-0302(99)75349-X 10386312

